# Developing an Intelligent System with Deep Learning Algorithms for Sentiment Analysis of E-Commerce Product Reviews

**DOI:** 10.1155/2022/3840071

**Published:** 2022-05-28

**Authors:** Mohammad Eid Alzahrani, Theyazn H. H. Aldhyani, Saleh Nagi Alsubari, Maha M. Althobaiti, Adil Fahad

**Affiliations:** ^1^Department of Engineering and Computer Science, Al Baha University, Al Bahah, Saudi Arabia; ^2^Applied College in Abqaiq, King Faisal University, P.O. Box 400, Al-Ahsa 31982, Saudi Arabia; ^3^Department of Computer Science & Information Technology, Dr. Babasaheb Amedkar Marathwada University, Aurangabad, India; ^4^Department of Computer Science, College of Computing and Information Technology, Taif University, P.O. Box 11099, Taif 21944, Saudi Arabia

## Abstract

Most consumers rely on online reviews when deciding to purchase e-commerce services or products. Unfortunately, the main problem of these reviews, which is not completely tackled, is the existence of deceptive reviews. The novelty of the proposed system is the application of opinion mining on consumers' reviews to help businesses and organizations continually improve their market strategies and obtain an in-depth analysis of the consumers' opinions regarding their products and brands. In this paper, the long short-term memory (LSTM) and deep learning convolutional neural network integrated with LSTM (CNN-LSTM) models were used for sentiment analysis of reviews in the e-commerce domain. The system was tested and evaluated by using real-time data that included reviews of cameras, laptops, mobile phones, tablets, televisions, and video surveillance products from the Amazon website. Data preprocessing steps, such as lowercase processing, stopword removal, punctuation removal, and tokenization, were used for data cleaning. The clean data were processed with the LSTM and CNN-LSTM models for the detection and classification of the consumers' sentiment into positive or negative. The LSTM and CNN-LSTM algorithms achieved an accuracy of 94% and 91%, respectively. We conclude that the deep learning techniques applied here provide optimal results for the classification of the customers' sentiment toward the products.

## 1. Introduction

Web 3.0 has the main features of the semantic web, artificial intelligence, connectivity, etc., allowing people to use social media to communicate and express their opinions about real-world events. In this context, the analysis of users' reviews is essential for companies to grow worldwide. This makes opinion mining a key player in the analysis of reviews and discussions. Nowadays, companies analyze this type of information to improve the quality and performance of the products and, consequently, survive in a competitive market. Opinion mining can be expressed as the reason behind any action or movement that people use to follow the reason [1].

Within the huge amount of data generated on the Internet, important information is hidden. Data mining techniques are used to extract information and solve various problems. Online product reviews have two important aspects under which data are stored on the Internet. Commercial websites are platforms where users express their sentiment or opinion on several topics. Sentiment analysis refers to a broad area of natural language processing (NLP), computational linguistics, and text mining [2]. The use of these techniques leads to the extraction and analysis of the opinion on a given product. Opinion mining defines an opinion as positive or negative, and sentiment analysis defines the polarity value of a user's opinion on a particular product or service. The current approaches of sentiment analysis are mainly [3] machine learning algorithms [4], lexicon-based methods, [5] and hybrid models [6, 7].

Negation is a prevalent morphological development that impacts polarity and, therefore, must be reflected in the assessment of sentiment. Automatic detection of negation in news articles is required for numerous text processing applications, including sentiment analysis. Here, we explored the role and importance of users' reviews concerning particular products on the decision using sentiment analysis. We present experimental results that demonstrate that sentiment analysis is appropriate to this end. The goal was to determine the polarity of the natural language of texts written in product reviews.

The existing straightforward approaches are statistical, based on frequencies of positive and negative words. Recently, researchers discovered new ways to account for other aspects of content, such as structural or semantic features. The present work focuses on the identification of document-level negation by using multiple computational methods. In recent years, with the exponential growth of smartphone use, many people are connected to social networking platforms, like Facebook, Twitter, and Instagram. Social networks have become a field to express beliefs or opinions, emotions, thoughts, personal issues, places, or personalities.

There are numerous studies applying sentiment analysis, some of which used real-time data from Twitter for extracting patterns by employing the Twitter-streaming application programming interface (API) [8, 9]. The sentiment analyzers are divided into two types: SentiWordNet [10] and WordNet [11]. Sentiment analysis uses positive and negative scores to classify opinions. By developing a model to analyze word sequence disambiguation [12], the Twitter-streaming API was used to gather data concerning the Indonesian presidential elections [13]. Needless tweets were removed, and the remaining data were investigated for sentimental aspects by dividing each tweet into numerous sub-tweets and calculating the sentiment polarity of the sub-tweets for predicting the consequence of the elections. The mean absolute error metric was used to evaluate the results, it noted that the prediction error was 0.6 better than the previous study [14]. To predict the Swedish election outcome by using Twitter data, a system was developed [15]. To predict the outcome of the European elections, a new method was designed that studied the similarity of the structure with the outcome of the vote. Another method was created to test Brazilian municipal elections in six cities [16]. In this methodology, sentiment analysis was applied along with a stratified sample [17] of users to compare the characteristics of the findings with the actual voters.

Many researchers have used machine learning and artificial intelligence to analyze the sentiment of tweets [18, 19]. In [20], the Naive Bayes, support vector machine (SVM) [21], and information entropy-based [22] models were applied to classify product reviews. A hybrid machine learning algorithm based on Twitter opinion mining was proposed in [23]. Heydari et al. [24] proposed time series model for fraudulent sentiment reviewer analysis. Hajek et al. [25] developed a deep feedforward neural network and convolution model to detect fake positive and negative review in an Amazon dataset. Long et al. [26] applied LSTM with multi-head attention network for predicting sentiment-based text using China social media dataset. Dong et al. [27] proposed supervised machine linear regression for predicting sentiment of customers presented in online shopping data using sentiment analysis learning approaches.

Researchers have been focusing on developing powerful models to deal with the ever-increasing complexity of big data [28, 29], as well as expanding sentiment analysis to a wide range of applications [30, 31], from financial forecasting to marketing strategies [32] among other areas [33, 34]. However, only a few of them analyzed different deep learning approaches to give real evidence of their performance [35]. Deep learning techniques are becoming increasingly popular. When assessing the performance of a single approach on a single dataset in a specific area, the results suggest that CNN and RNN have relatively good accuracy. Based on AdaBoost combination, Gao et al. [36] proposed CNN model for sentiment analysis in user-generated text. In this vein, Hassan and Mahmood [37] demonstrated that the CNN and RNN models overcame the problem of short texts in deep learning models.

Some traditional approaches, which are assisted by machine learning techniques, are based on aspects of the used language. Using the domain of movie opinions, Pang et al. [18] studied the performance of various machine learning algorithms, including Naive Bayes, maximum entropy, and SVM. By using SVM with unigrams, they achieved an accuracy of 82.9%. NLP is typically used to extract features used by a sentiment classifier. In this aspect, the majority of NLP strategies are centered on the usage of n-grams but the use of a bag-of-words strategy is also common [38, 39]. Numerous studies have demonstrated significant results when employing the bag-of-words as a text representation for item categorization [40–44].

Researchers have taken advantage of NLP themes to develop deep learning models based on neural networks with more than three layers, according to the journal Nature. Most of these studies found that deep learning models accurately detect sentiment in various situations. The CNN [45, 46], RNN [47], deep neural network [48], recursive neural deep model [49], and the attention-based bidirectional CNN-RNN [50] models are some representative examples. Some researchers combine models, which are then referred to as hybrid neural networks. The hierarchical bidirectional RNN is an example of a hybrid neural network [51]. The main issue with sentiment analysis of product reviews in the e-commerce domain is the existence of fake reviews that lead customers to select undesired products [52].

The main contributions of the proposed research are the following:

1. The generation of a sentiment score using a lexicon-based approach for each product review of the dataset.
2. Labeling the review texts as negative if the generated sentiment score is <0 or positive if the score is >1.
3. The combination of all product reviews into a single data frame to obtain more sentiment-related words.
4. Improving the accuracy by developing a hybrid deep learning model combining the CNN and LSTM models for the product-related sentiment classification.
5. Comparing the classification performance of the CNN-LSTM and LSTM models.

## 2. Materials and Methods

**Figure 1:**
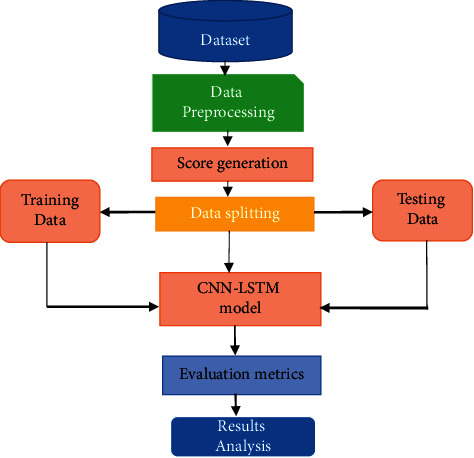
Framework for the proposed methodology.

The proposed methodology for predicting the review-related sentiments is based on the deep learning algorithms presented here. The phases of the proposed system are the following: dataset collection, data preprocessing, generating the sentiment score, polarity calculation, applying the CNN-LSTM model, evaluation metrics, and analysis of the results.  Figure 1 shows the framework of the proposed methodology used in the present study.

### 2.1. Datasets

To evaluate the proposed system, the dataset [53] was collected from reviews on the Amazon website in JSON file format. Each JSON file comprises a number of reviews (Table 1). The dataset includes reviews of laptops, mobile phones, tablets, televisions, and video surveillance products. The data preprocessing includes various steps, such as lowercase processing with meta-features like the reviewer's ID, the product ID, and the review text.

### 2.2. Data Preprocessing

We implemented different preprocessing steps aiming at cleaning the review texts so that they are easy to process. The following preprocessing methods were performed on the dataset as a whole.

#### 2.2.1. Lowercase

It entails converting whole words of the review text into lowercase words.

#### 2.2.2. Stopword Removal

Stopwords are widely used words in a language, such as “the,” “a,” “an,” “is,” and “are”. As these words do not carry any information significant for the model, they were removed from the content of the review.

#### 2.2.3. Punctuation Removal

All punctuation marks in the review texts were removed.

#### 2.2.4. One-Word Review Elimination

Reviews that included only one word were eliminated.

#### 2.2.5. Contraction Removal

This process replaces a word originally written in the short form with the respective full form; for instance, “when've” becomes “when have.”

#### 2.2.6. Tokenization

Each sentence of the review texts was divided into small pieces of words or tokens.

#### 2.2.7. Part-of-Speech Tagging

This step is used to tag each word present in the sentence with a POS tag, for example, “VB” for a verb, “AJJ” for an adjective, and “NN” for a noun.

#### 2.2.8. Score Generation

The review text was evaluated for sentiment, and a score was generated. For calculating the sentiment score, the dataset was matched with opinion lexicon [53] that consists of 5,000 positive words and 4,500 negative words with their respective scores. The sentiment score was calculated for each review text based on the scores of the lexicon. The review text was labeled as positive if the score was >0; otherwise, it was labeled as negative.

#### 2.2.9. Word Embeddings

We calculated numerical vectors with every preprocessed sentence in the product review dataset using the “Word embeddings” method. To create word indices, we first turned all of the review text terms into sequences. The Keras text tokenizer [54] is being used to obtain those indices. We made sure that no term or word gets a zero index in the tokenizer, and that the vocabulary size is adjusted properly. Then, for each single word in the training and testing sets, a distinctive index is generated, which is employed to create numeric vectors of all review texts of the dataset.

### 2.3. The CNN-LSTM Model

**Figure 2:**
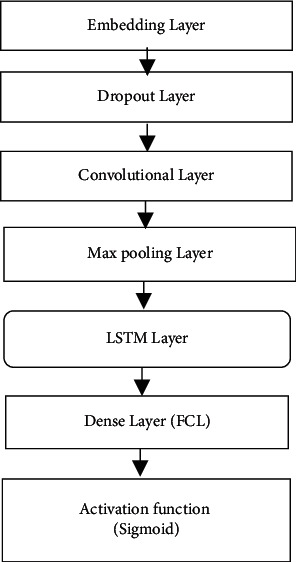
The structure of the CNN-LSTM model.

Figure 2 presents the structure of the CNN-LSTM model used for sentiment classification of customers' reviews using an Amazon dataset.

#### 2.3.1. Embedding Layer

This is the initial layer of the CNN-LSTM model that is used to transform each word in the training dataset into an actual-valued vector, meaning that a set of sentiment-related words are constructed and transformed into a numerical form. This process is known as word embedding. The embedding layer consisted of three components: the vocabulary size (maximum features; 15,000 words), the embedding dimensions (50), and the input sequence length (400 words).

#### 2.3.2. Dropout Layer

The main task of this layer is to avoid the overfitting of the model [52]. Here, we assigned the value 0.4 to the dropout rate parameter, where this value has a range between 0 and 1. The main function of the dropout layer is to arbitrarily deactivate a set of neurons in the embedding layer, where every neuron denotes the dense exemplification of a sentiment word in a review text.

CNN is a deep learning technique used in different areas such as natural language preprocessing tasks, computer vision, and medical image processing.

#### 2.3.3. Convolution Layer

The third layer of the CNN-LSTM model is used for the extraction of features from the input matrix. It uses *n* convolution filters that operate over the elements of the input sequence matrix to find the convolutions for each sequence. We set the number of filters to 64 and the size of the filter kernel to  3 × 3.

#### 2.3.4. Max Pooling Layer

This layer performs downsampling beside the spatial dimensionality of the given input sequences. It considers the maximum value of all input features in the pool of each filter kernel. It has assigned to 5 × 5 kernel.

#### 2.3.5. LSTM Layer

**Figure 3:**
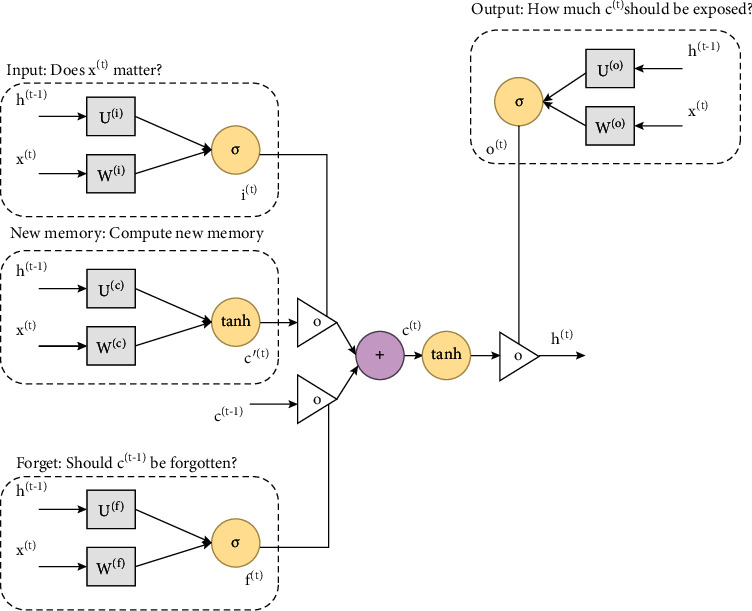
The structure of the LSTM model.

LSTM is a type of RNN capable of learning long-term dependence [52]. We used an LSTM layer and assigned it to 50 hidden units toward the next layer. One of the most notable advantages of employing a convolutional neural network as feature extraction technique beyond a traditional LSTM is the reduction in the aggregating amount of features. Throughout the feature extraction process, a sentiment classification model uses these features (words) for prediction of the product review text as positive or negative sentiment. LSTM executes precalculations for the input sequences before providing an output to the last layer of the network. In every cell, four discrete computations are conducted based on four gates: input (*i*_*t*_), forget (*f*_*t*_), candidate (*c*_*t*_), and output (*o*_*t*_). The structure of the LSTM model is presented in Figure 3. The equations for these gates are as follows:

(1) $$\begin{array}{c} f_{t}=\text{sig}\left(Wf_{xt}+Uf_{ht}-1+b_{f}\right),\\ i_{t}=\text{sig}\left(Wi_{xt}+Ui_{ht\;}-1+\;b_{i}\right),\\ O_{t}=\text{sig}\left(Wo_{xt}+Uo_{ht}-1+b_{o}\right),\\ c∼t=\tanh\left(wc_{xt}+Uc_{ht}-1+bc\right),\\ C_{t}=\left(f_{to}ct-1+i_{to}c∼t\right),\\ h_{t}=O_{to}*\;\tanh\left(C_{t}\right),\\ \tanh\left(x\right)=\;\frac{\;1-e^{2x}}{1-e^{2x}}, \end{array}$$

where sig and tanh are the sigmoid and tangent activation functions, respectively, *X* is the input data, W and *b* represent the weight and bias factor, respectively, *C*_*t*_  is the cell state,  *c* ~ *t* is the candidate gate, and  *h*_*t*_  refers to the output of the LSTM cell.

#### 2.3.6. Dense Layer (Fully Connected Layer)

This is a hidden layer in the CNN-LSTM model. It consists of 512 artificial connected neurons that connect all neurons of the network. The function applied to this layer is the rectified linear unit described by the following equation:

(2) $$\begin{array}{c} f\left(x\right)=\max\left(o,x\right). \end{array}$$

#### 2.3.7. Sigmoid Activation Function

It is the first layer that detects and classifies the output classes (positive or negative sentiment). The sigmoid function formula is given as follows (Algorithm 1):

(3) $$\begin{array}{c} \sigma \;=\;\frac{\;1}{1-e^{2x}}. \end{array}$$

### 2.4. Evaluation Metrics

To evaluate the proposed models (CNN-LSTM and LSTM), the accuracy, precision, recall, F1-score, and specificity metrics were used. The performance measurements are presented below:

(4) $$\begin{array}{c} \text{Accuracy}=\frac{\text{TP}+\text{TN}}{\text{FP}+\text{FN}+\text{TP}+\text{TN}}\times 100\%,\\ \text{Precision}=\frac{\text{TP}}{\text{TP}+\text{FP}}\;\times 100\%,\\ \text{F}1-\text{score}=2*\frac{\text{precision}\times \text{sensitivity}}{\text{precision}+\text{sensitivity}}\;\times 100\%,\\ \text{Specificity}=\frac{\text{TN}}{\text{TN}+\text{FP}}\;\times 100\%,\\ \text{Recall}=\frac{\text{TP}}{\text{TP}+\text{FN}}\;\times 100\%, \end{array}$$

where true positive (TP) represents the total number of samples that are successfully classified as positive sentiment, false positive (FP) is the total number of samples that are incorrectly classified as negative sentiments, true negative (TN) denotes the total number of samples that are successfully classified as negative sentiment, and false negative (FN) represents the total number of samples that are incorrectly classified as positive sentiments.

## 3. Experimental Results

**Figure 4:**
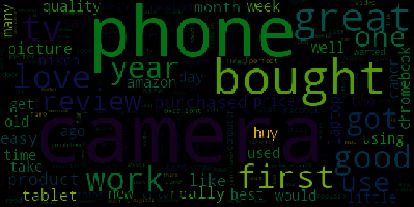
Word cloud of the dataset.

In this section, we present the experimental results of the application of the CNN-LSTM and LSTM models for the analysis and prediction of sentiment in the e-commerce domain. We used hardware with 4 GB RAM and an i7 2800 CPU and ran the experiments on the Jupyter environment. The evaluation metrics (accuracy, precision, F1-score, recall, and specificity) were employed to examine the proposed system. The word cloud (sentiment words and product names) of the dataset is presented in Figure 4, which shows graphical representations of words (large font words) that give greater importance to that seem more repeatedly in the used product review dataset.

### 3.1. Data Splitting

In this phase, we divided the dataset that consisted of 13,057 product reviews into 70% training, 10% validation, and 20% testing datasets. Then, the CNN-LSTM and LSTM models were applied to detect and classify the review texts into positive or negative.  Table 2 shows the splitting of the dataset.

### 3.2. Results and Discussion

Table 3 shows the results of the deep learning approaches. The CNN-LSTM model achieved high accuracy (96%).

**Figure 5:**
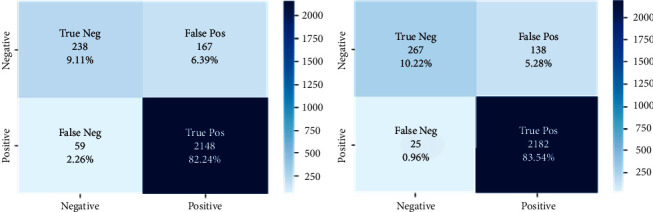
Confusion matrix of the (a) LSTM and (b) CNN-LSTM models.

The confusion matrix of the CNN-LSTM and LSTM models is shown in Figure 5. The confusion matrix is used to present the rates of TP, FP, TN, and FN of the sample. Based on these rates, the evaluation metrics (specificity, accuracy, recall, precision, and F1-score) were calculated to evaluate the CNN-LSTM model using unseen data to predict the sentiment of customers. LSTM resulted in 82.24% TP, while CNN-LSTM resulted in 83.54% TP. As for misclassification, LSTM resulted in 6.39% FP and CNN-LSTM in 5.28% FP, indicating that the CNN-LSTM model was slightly better than the LSTM model.

**Figure 6:**
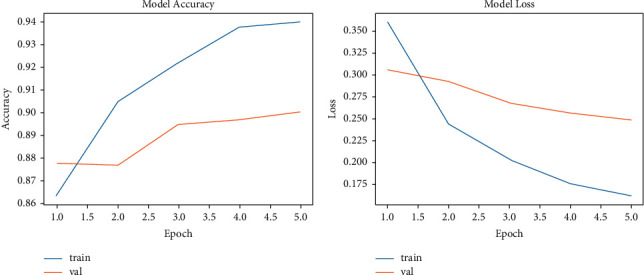
The performance of the LSTM model: (a) accuracy and (b) loss.

The accuracy performance of LSTM for the training and validation datasets is presented in Figure 6. The LSTM model presented increasing accuracy during the training phase (from 86% to 94%), whereas in the testing phase, it achieved 91% accuracy with 10 epochs. The loss of the LSTM model in the training phase decreased from 5 to 0.35, while in the validation phase, the model loss decreased from 0.3 to 0.27.

**Figure 7:**
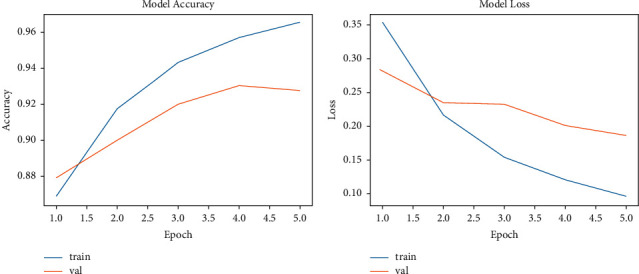
The performance of the CNN-LSTM model: (a) accuracy and (b) loss.

The accuracy performance of the CNN-LSTM during the training phase increased from 87.50% to 97%. In the validation phase, the accuracy performance reached 94% (Figure 7(a)). The loss of the CNN-LSTM model in the validation phase was 0.20 (Figure 7(b)).

The dataset developed by Rajkumar et al. [53] proposed SVM and Naive Bayes methods to predict sentiment analysis. They collected data from Amazon concerning mobile phones, tablets, cameras, and televisions. They applied the SVM method to each dataset individually. Here, we applied deep learning models to all the datasets combined. The empirical results of our system were compared with the results of [28] and are shown in Table 4. The CNN-LSTM model achieved an accuracy of 94%.

## 4. Conclusion

Recently, sentiment analysis has become a valuable tool for the generation and evaluation of different types of data, helping the decision-making processes that lead to the improvement of businesses and companies. Social networking creates a large amount of data that require processing and analysis to obtain relevant insights. In the present study, the experimental dataset was collected from the Amazon website and included reviews of laptops, mobile phones, tablets, televisions, and video surveillance products. The lexicon-based approach was used for the calculation of the sentiment score for each review text. The output of the preprocessed data was classified with the LSTM and CNN-LSTM models. The experimental results showed that our model was satisfactory in all the measurement metrics.

## Figures and Tables

**Algorithm 1:**
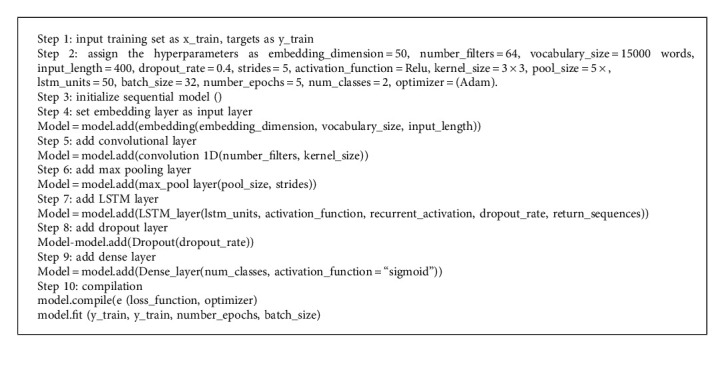
CNN-LSTM.

**Table 1:** The number of reviews per product category.

Product name	Review count
Laptops	1,946
Mobile phones	1,918
Tablets	1,894
Televisions	1,596
Video surveillance products	2,597

**Table 2:** The splitting of the dataset.

Total number of reviews	Training set 80%	Validation set 10%	Testing set 20%
13,057 (11,184 positive; 1,873 negative)	9,400	1,045	2,612

**Table 3:** Results of the deep learning models.

Models	Specificity	Accuracy (%)	Precision (%)	Recall (%)	F1-score (%)
LSTM	95	91.03	92.07	97.73	95.50
CNN-LSTM	96	94	94	99	96.03

**Table 4:** Significant results of the CNN-LSTM model compared to the SVM method.

Models	Datasets	Accuracy (%)
Support vector machine [28]	Televisions, tablets, mobile phones, laptops, and video surveillance	88, 84, 92, 88, and 93
Proposed system (CNN-LSTM)	All dataset	94

## Data Availability

We have collected dataset from authors of research article: https://www.researchgate.net/publication/326985579_ Sentiment_Analysis_ on_Product_Reviews_Using_ Machine _Learning_ Techniques_Proceeding_of_CISC_ 2017
